# Supplementary material for: FERN – a Java framework for stochastic simulation and evaluation of reaction networks
Source: BMC Bioinformatics. 2008 Aug 29;9:356. doi: 10.1186/1471-2105-9-356 (PMC2553347; doi:10.1186/1471-2105-9-356)
Supplement: Additional file 1 — FERN distribution, Version 1.3. This archive contains the FERN source code and binaries as well as documentation and example models in FernML and SBML. [file 1471-2105-9-356-S1.zip › fern/doc/javadoc/index-files/index-5.html]

E-Index


---


|  |  |  |
| --- | --- | --- |
| **Overview** \| Package \| Class \| Use \| **Tree** \| **Deprecated** \| **Index** \| **Help** | |  |
| **PREV LETTER**   **NEXT LETTER** | **FRAMES**    **NO FRAMES**     **All Classes** |  |


A B C D E F G H I J L M N O P R S T U V 

---


## **E**

**end()** - Method in class fern.benchmark.Benchmark: Gets the elapsed time since the last call of `start` in nanoseconds **ExamplePath** - Class in fern.example: Determine the path to the example network files. **ExamplePath()** - Constructor for class fern.example.ExamplePath: **exists(String)** - Static method in class fern.example.ExamplePath: **ExtendedPane** - Class in fern.cellDesigner.ui: **ExtendedPane(MainFrame)** - Constructor for class fern.cellDesigner.ui.ExtendedPane: **ExtendedPane** - Class in fern.cytoscape.ui: **ExtendedPane(MainFrame)** - Constructor for class fern.cytoscape.ui.ExtendedPane: **ExtractSubNetwork** - Class in fern.network.modification: Extracts some reactions / species from a given net to form a new network. **ExtractSubNetwork(Network, BitVector, BitVector)** - Constructor for class fern.network.modification.ExtractSubNetwork: Extracts a given subnet from a network.

---


|  |  |  |
| --- | --- | --- |
| **Overview** \| Package \| Class \| Use \| **Tree** \| **Deprecated** \| **Index** \| **Help** | |  |
| **PREV LETTER**   **NEXT LETTER** | **FRAMES**    **NO FRAMES**     **All Classes** |  |


A B C D E F G H I J L M N O P R S T U V 

---
